# Zika Virus-Encoded NS2A and NS4A Strongly Downregulate NF-κB Promoter Activity

**DOI:** 10.4014/jmb.2011.11003

**Published:** 2020-11-17

**Authors:** Jeong Yoon Lee, Thi Thuy Ngan Nguyen, Jinjong Myoung

**Affiliations:** Korea Zoonosis Research Institute, Department of Bioactive Material Science and Genetic Engineering Research Institute, Jeonbuk National University, Jeonju 54531, Republic of Korea

**Keywords:** Zika virus, interferon, NS2A, NS4A

## Abstract

Since Zika virus (ZIKV) was first detected in Uganda in 1947, serious outbreaks have occurred globally in Yap Island, French Polynesia and Brazil. Even though the number of infections and spread of ZIKV have risen sharply, the pathogenesis and replication mechanisms of ZIKV have not been well studied. ZIKV, a recently highlighted Flavivirus, is a mosquito-borne emerging virus causing microcephaly and the Guillain-Barre syndrome in fetuses and adults, respectively. ZIKV polyprotein consists of three structural proteins named C, prM and E and seven nonstructural proteins named NS1, NS2A, NS2B, NS3, NS4A, NS4B, and NS5 in an 11-kb single-stranded positive sense RNA genome. The function of individual ZIKV genes on the host innate immune response has barely been studied. In this study, we investigated the modulations of the NF-κB promoter activity induced by the MDA5/RIG-I signaling pathway. According to our results, two nonstructural proteins, NS2A and NS4A, dramatically suppressed the NF-κB promoter activity by inhibiting signaling factors involved in the MDA5/RIG-I signaling pathway. Interestingly, NS2A suppressed all components of MDA5/RIG-I signaling pathway, but NS4A inhibited most signaling molecules, except IKKε and IRF3-5D. In addition, both NS2A and NS4A downregulated MDA5-induced NF-κB promoter activity in a dosedependent manner. Taken together, our results suggest that NS2A and NS4A signifcantly antagonize MDA5/RIG-I-mediated NF-κB production, and these proteins seem to be controlled by different mechanisms. This study could help understand the mechanisms of how ZIKV controls innate immune responses and may also assist in the development of ZIKV-specific therapeutics.

## Introduction

Zika virus (ZIKV) belongs to the genus *Flavivirus* of the family *Flaviviridae*. The genome of ZIKV is 11-kb, single-stranded positive sense RNA encoding three structural proteins: the capsid (C), precursor membrane protein (prM) and envelope (E), and seven nonstructural proteins: NS1, NS2A, NS2B, NS3, NS4A, NS4B, and NS5 [1].

In 1947, ZIKV was identified from a febrile sentinel rhesus monkey (Rhesus 766) at the Zika Forest in Uganda [2]. Historically, serious outbreaks have occurred in Yap Island in 2007, French Polynesia in 2013 and in Central and South America and the Caribbean regions in 2015 [3-5]. Mild symptoms including fever, rash, conjunctivitis and arthralgia and severe symptoms such as microcephaly and Guillain-Barre syndrome in fetuses and adults, respectively, have been reported to be caused by ZIKV infection [6-9]. Two *Aedes* mosquitoes, *Aedes aegypti* and *Aedes albopictus*, are recognized as transmitters to humans [1]. Other transmission routes of ZIKV have also been reported in some cases such as mother to fetus, breastfeeding, sexual intercourse or blood transfusion [10].

Nuclear factor kappa-light-chain-enhancer of activated B cells (NF-κB) is a protein complex related to DNA transcription, cytokine production and cell survival. Activation of NF-κB has been reported to play a pivotal role in lots of cellular responses: stress, cytokines, immune responses to pathogens such as bacteria and viruses, and external environment factors [11-14]. NF-κB plays an important role in the immune response to pathogen infection. Disregulation of NF-κB is involved in cancer, chronic inflammatory diseases and pathological development in viral infections [15].

NF-κB is one of crucial factors in the immune responses against virus infection [16-22]. During RNA virus infection, the virus genome is mainly recognized by pattern recognition receptors (PRRs): melanoma differentiation associated gene 5 (MDA5) and retinoic acid-inducible gene 1 (RIG-I) as well as the laboratory of genetics and physiology 2 (LGP2) [23-25]. Upon recognition of a virus genome, the PRR pathway is activated, resulting in expression of type I interferon (IFN) [17,26-30]. Consequently, the production of IFN induces pro-inflammatory cytokines by stimulating NF-κB [21, 31]. Also, type I IFN production is induced by the signal cascade of the mitochondrial antiviral adaptor protein (MAVS), inhibitor of kappa-B kinase epsilon (IKKε), TANK binding kinase 1 (TBK1), and interferon regulatory factor 3 (IRF3) of the MDA5/RIG-I pathway. Furthermore, although the canonical NF-κB pathway involving IKKα, IKKβ, p50 and p60 is widely known, the non-canonical NF-κB pathway is also known to be involved in MDA5/RIG-I pathway to produce type I IFNs by activating the IFN-stimulated response element (ISRE) and IFN-stimulated genes (ISGs), which protect the host cells against virus replication.

In this study, we found that the ZIKV NS2A and NS4A proteins are major viral proteins antagonizing NF-κB production. NS2A and NS4A suppressed NF-κB promoter activities through not only RIG-I-like receptors (RLRs) such as MDA5 and RIG-I but also many other signaling molecules in the MDA5/RIG-I signaling pathway. In addition, ZIKV C and prM contributed to reduction of NF-κB production by the inhibition of MDA5, constitutively active RIG-I (RIG-I-1-228) and TBK1. Also, ZIKV NS1 displayed activities to downregulate NF-κB production via the inhibition of RIG-I-1-228, TBK1 and full-length IRF3 (IRF3-FL). ZIKV E and NS2B-NS3 are also associated with repression of NF-κB production through downregulation of RIG-I-1-228 and TBK1. Some other ZIKV proteins seems to act as mild antagonists of NK-kB promoter activation as well as NS2A and NS4A. Understanding the modulation of host immunity controlled by ZIKV will provide important clues to develop antiviral and preventive therapeutics.

## Materials and Methods

### Cells

Human embryonic kidney 293T (HEK293T) cells were bought from the American Type Culture Collection (ATCC) and grown in Dulbecco’s modified Eagle’s medium (DMEM) (Welgene, Republic of Korea) with 10% fetal bovine serum (FBS) (Welgene) and 1% penicillin-streptomycin (Gibco, USA). The cells were maintained at 37°C, in a 5% CO_2_ humidifying incubator. Dulbecco’s phosphate-buffered saline (DPBS) and 0.05% Trypsin-EDTA purchased from Welgene were also used.

### Antibodies

A mouse monoclonal anti-FLAG antibody (M2) (Millipore Sigma, USA) was used in 1:5000 dilution. anti-HA (6E2) mouse monoclonal antibody, horseradish peroxidase (HRP)-conjugated rabbit monoclonal anti-GAPDH (14C10), HRP-conjugated anti-rabbit IgG antibody and HRP-conjugated anti-mouse IgG antibody (Cell Signaling, USA) were utilized in 1:1000, 1:2000, 1:2000 and 1:2000 dilution, respectively.

### Reagents

Opti-minimal essential medium (MEM) (Gibco), polyethylenimine (PEI; Millipore Sigma) were used for plasmid transfection and cOmplete Mini Protease Inhibitor Cocktail (Millipore Sigma) was purchased for luciferase assay and western blotting assay. The luciferase assay and Beta-Glo assay systems (Promega, USA), 4 × Laemmli sample buffer and 2-mercatoethanol (Bio-Rad, USA), Amersham ECL Prime western blotting detection reagent, Amersham ECL western blotting detection reagent, and Amersham Protran 0.45 nitro-cellulose (NC) blotting membrane (GE Healthcare Life Sciences, USA) were obtained. Restriction enzymes including EcoRI-HF, BamHI-HF, NotI-HF, NheI-HF, PmeI, PmlI, XhoI and XbaI, and T4 DNA ligase were from New England Biolabs (NEB, USA). MAX Efficiency DH5α competent cells (Thermo Fisher Scientific, USA) and Pfu Plus DNA polymerase (Elpisbio, Republic of Korea) were purchased.

### Plasmids

The methods for generating the pcDNA3.1-Neo-JY3 vector and pcDNA3.1-Neo-JY3-3xFLAG-N vector have been previously described [20, 27, 32]. All constructs utilized in this study were fully accounted in the previous paper [33-35]. Reverse transcription-quantitative polymerase chain reaction (RT-qPCR) to amplify ZIKV genes were previously described in detail [30].

### Transfection and Luciferase Assay

Detailed experimental procedures were described elsewhere [33-35]. Briefly, HEK293T 8 × 10^5^ cells/well were prepared in a 6-well plate. After 24 h, 500 ng of interferon (IFN)-β-luc plasmid, 100 ng of β-gal-expression plasmid, 500 ng of each immune gene expression plasmid and 1,000 ng of individual ZIKV gene-encoding plasmid were used for transfection utilizing the PEI reagent at a ratio of 1: 2 (DNA: PEI). At 24 h post-transfection (hpt), samples were collected using lysis buffer with the protease inhibitor cocktail and centrifuged at 15,000 ×*g*, 4°C, for 15 min to remove lysed cells. Then, 25 μl of the sample supernatants and 25 μl assay substrate were mixed and applied to either the luciferase assay or Beta-Glo assay according to the manufacturer’s instructions. Firefly luciferase activities normalized by the internal control (β-gal) were used to calculate the fold changes over the empty vector control. Paired two-tailed Student’s *t*-test was conducted and *p* < 0.05 was considered as statistically significant.

## Results

### Both MDA5- and RIG-I-Induced NF-κB Activation Were Strongly Inhibited by ZIKV-Encoded NS2A and NS4A Proteins

**Fig. 1:**
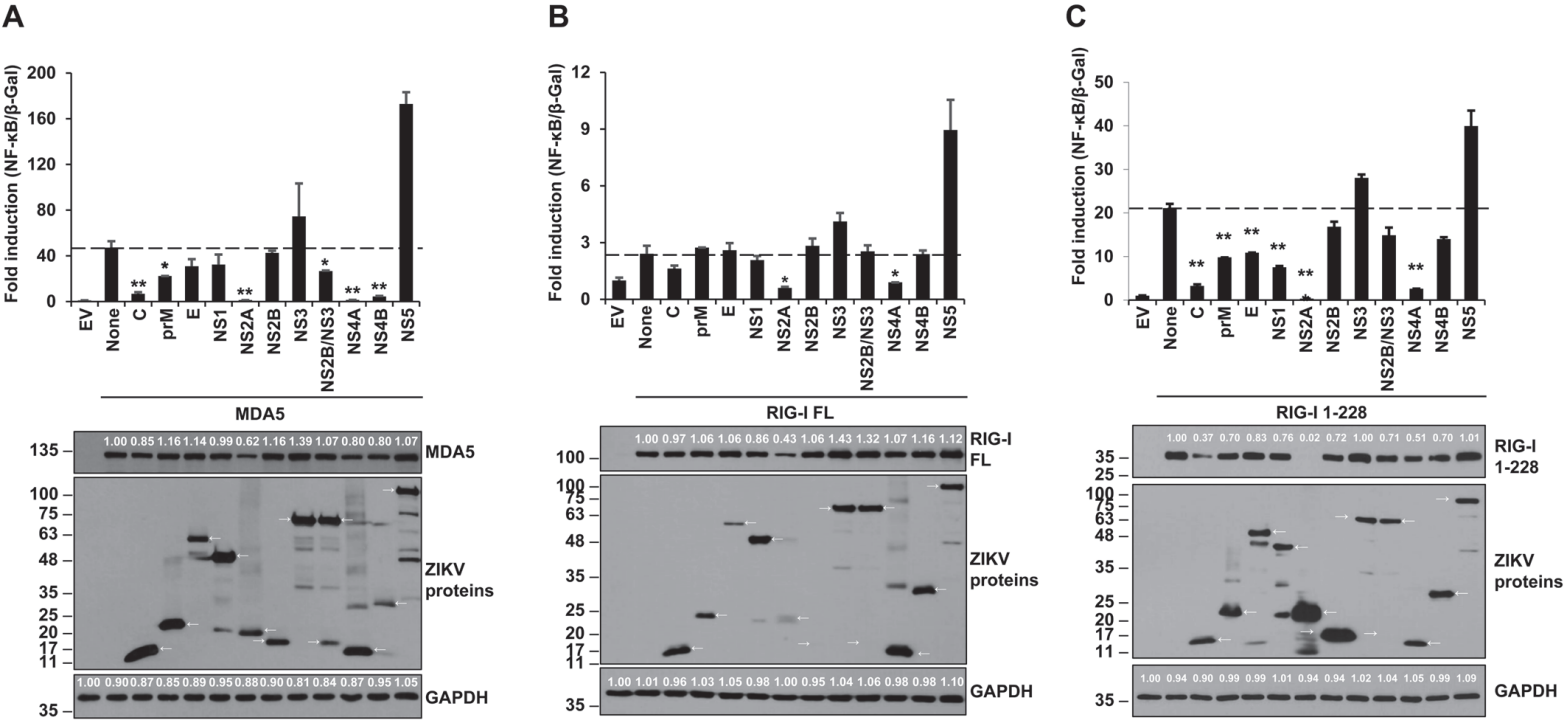
MDA5 and RIG-I, the cytoplasmic RNA sensors, were antagonized by ZIKV-encoded proteins. Plasmids encoding individual ZIKV protein, NF-κB-luc, β-Gal, and MDA5 (**A**), RIG-I FL (**B**), and RIG-I 1-228 (**C**) were co-transfected into HEK293T cells. At 24 hpt, the lysed transfected cells were applied to luciferase assay (top panel) and western blotting (bottom panel). Anti-HA antibody (for MDA5, RIG-I FL, and RIG-I 1-228), anti-FLAG antibody (for ZIKV proteins), and anti-GAPDH antibody (for human GAPDH) were used for western blotting assay. Representative data are shown from two independent experiments. Student’s two-tailed *t*-test were applied for statistical significance, **p < 0.01, *p < 0.05. The sizes of the proteins are presented on the left (kDa) and arrows indicate the expected proteins.

The signaling pathways of the cytoplasmic dsRNA sensors MDA5 and RIG-I were tested to find out the possible targets of ZIKV-encoded genes influencing NF-κB promoter activity (Fig. 1). Each plasmid encoding full-length MDA5, full-length RIG-I or constitutively active RIG-I-1-228 was co-transfected into HEK293T cells with NF-κB-luc, β-gal and each individual ZIKV gene-expressing plasmid. At 24 hpt, luciferase assays and β-gal assays were performed. In the results, ZIKV NS2A and NS4A significantly downregulated the NF-κB promoter activities activated by all forms of RLRs (MDA5, RIG-I and RIG-I-1-228) (Fig. 1). ZIKV C, prM, NS2A and NS4A proteins commonly reduced NF-κB production in all targets, MDA5, RIG-I and RIG-I-1-228 (Figs. 1A-1C). However, NS2B on MDA5 signaling pathway and E and NS1 on RIG-I-1-228 related signaling pathway negatively affected NF-κB production. These results suggest that NF-κB promoter activity induced by MDA5/RIG-I signaling pathway was dramatically reduced by these ZIKV-encoded proteins.

### ZIKV-Encoded NS2A and NS4A Proteins Significantly Inhibit MAVS-, TBK1-, and IKKε-Induced NF-κB Promoter Activity

**Fig. 2:**
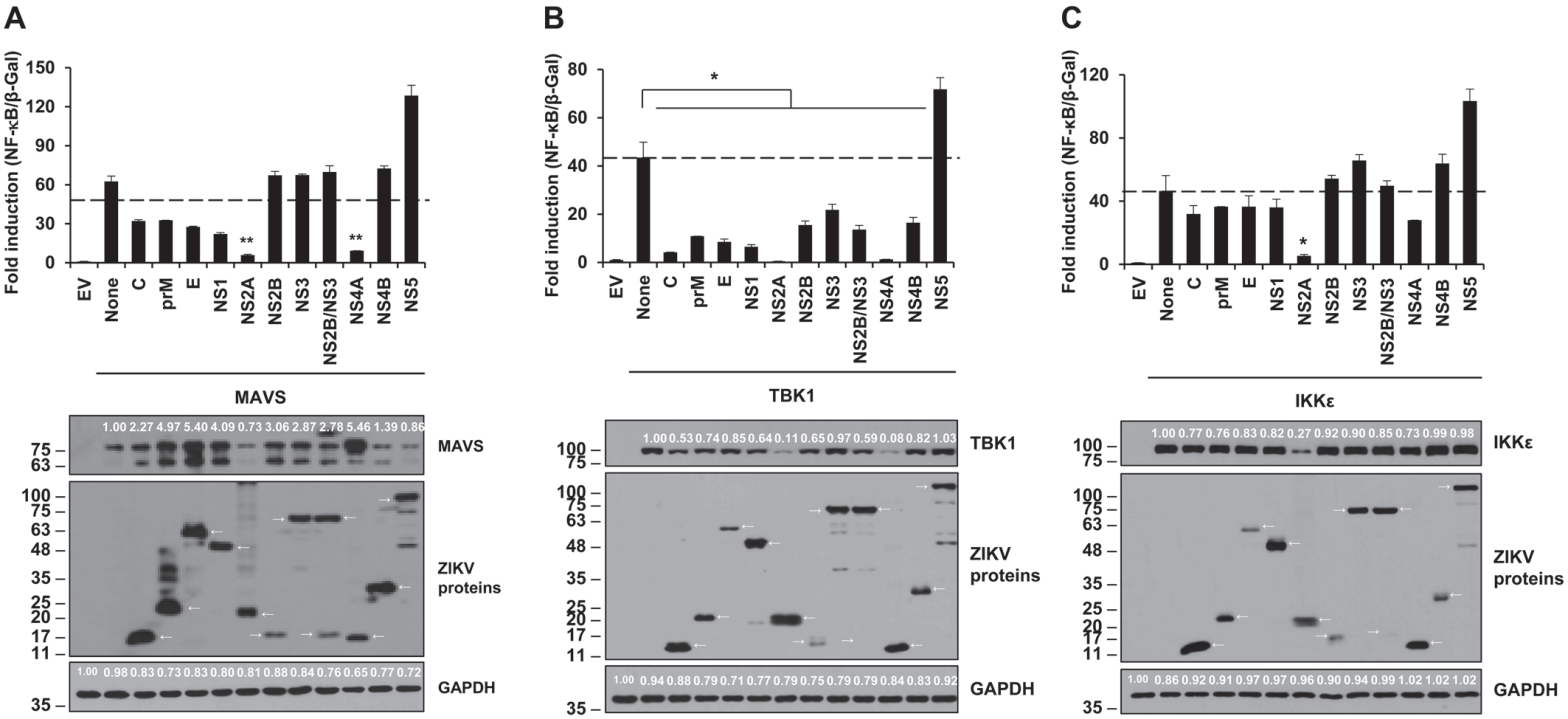
Induction of the NF-κB promoter by MAVS, TBK1 and IKKε were inhibited by ZIKV-encoded proteins. Plasmids encoding individual ZIKV protein, NF-κB-luc, β-Gal, and MAVS (**A**), TBK1 (**B**), or IKKε (**C**) were co-transfected to HEK293T cells. At 24 hpt, the activities of NF-κB promoter were analyzed by for the luciferase assay (top panel) and western blotting (bottom panel). Anti-MAVS antibody or anti-HA antibody (for TBK1 and IKKε), anti-FLAG antibody (for ZIKV proteins), or anti-GAPDH antibody (for human GAPDH) were used. Representative data are shown from two independent experiments. Student’s two-tailed *t*-test were applied for statistical significance, **p < 0.01, *p < 0.05. The sizes of the proteins are presented on the left (kDa) and arrows indicate the expected proteins.

We screened to determine which ZIKV proteins can hinder NF-κB promoter activity at the level of MAVS, TBK1, and IKKε, which are the downstream proteins of MDA5 and RIG-I. The MAVS-, TBK1- or IKKε-expressing plasmids were co-transfected with individual ZIKV gene, and then, at 24 hpt, the NF-κB promoter activity was measured by luciferase assay (Fig. 2). Interestingly, NS2A and NS4A showed dramatic inhibition on MAVS-, TBK1-, and IKKε-induced NF-κB promoter activities except NS4A on IKKε showing moderate inhibition. Targeting of MAVS and IKKε by ZIKV NS4A inhibited NF-κB promoter activity without influencing protein expression levels of those proteins (Figs. 2A and 2C). The downregulation of TBK1 production seemed to be associated with all ZIKV proteins except NS5 (Fig. 2B). Even though the data showed all ZIKV proteins critically suppressed TBK1-related NF-κB promoter activation, NS5 led to an increase of TBK1-mediated NF-κB production (Fig. 2B). Although both NS2A and NS4A are shown to inhibit MAVS-mediated NF-κB promoter activity, the observation that levels of MAVS affected only by NS4A was unexpected (Fig. 2A).

### ZIKV NS2A Leads to Significant Inhibition of the NF-κB Promoter Activity by all IRF3 Forms (IRF3-FL, IRF3-1-390, and IRF3-5D)

**Fig. 3:**
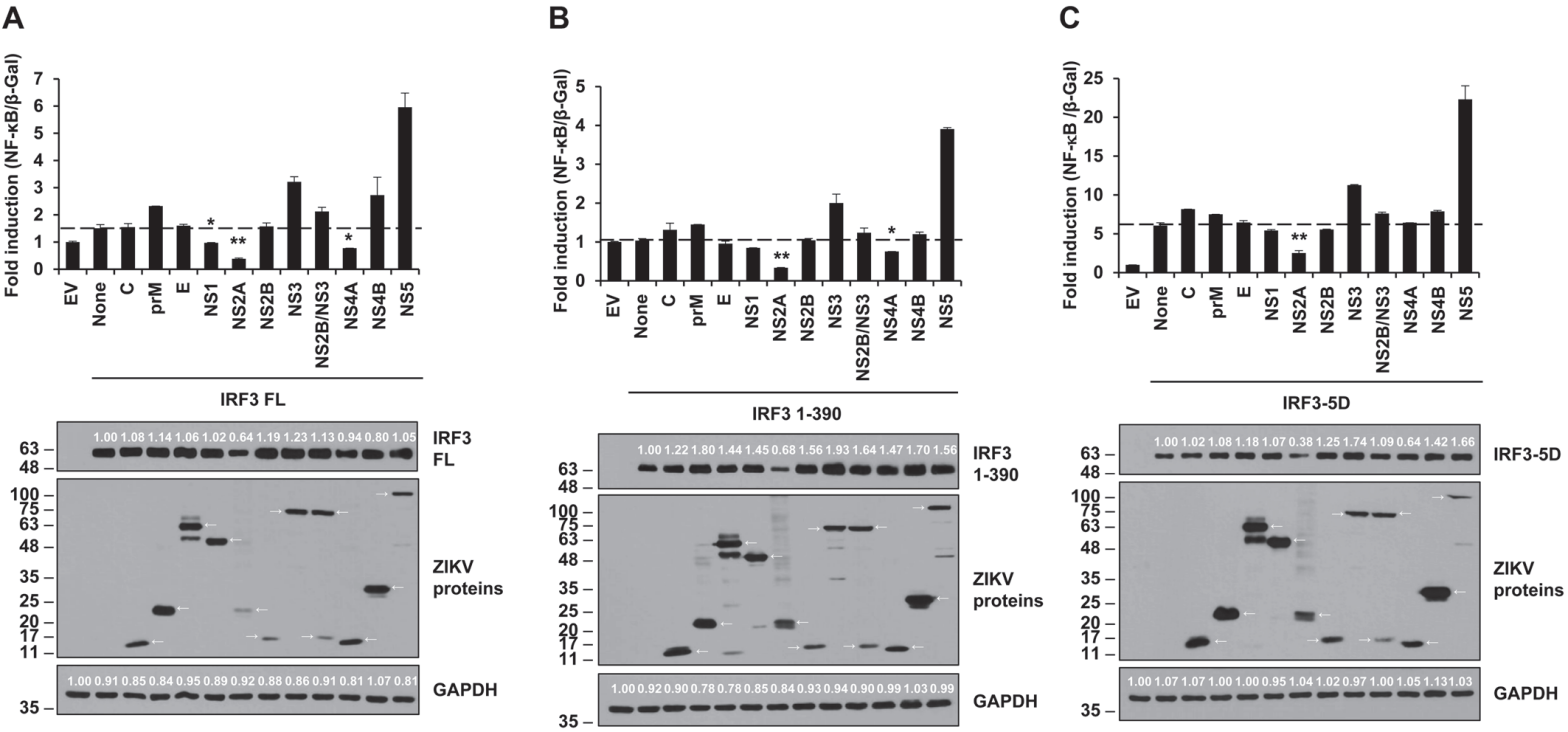
ZIKV NS2A and NS4A impeded activation of the NF-κB promoter by IRF FL, IRF3 1-390, and IRF3-5D. Various plasmids encoding individual ZIKV protein, NF-κB-luc, β-Gal, and IRF3 FL (**A**), IRF3 1-390 (**B**), IRF3-5D (**C**) were co-transfected to HEK293T cells. After 24 hours, the lysed cells were collected for luciferase assay (top panel) and western blotting (bottom panel) using anti-HA antibody (for IRF3 FL, IRF3 1-390, and IRF3-5D), anti-FLAG antibody (for ZIKV proteins), and anti-GAPDH antibody (for human GAPDH). Representative data from two independent experiments were presented. Student’s two-tailed *t*-test were applied for statistical significance, **p < 0.01, *p < 0.05. The sizes of the proteins are presented on the left (kDa) and arrows indicate the expected proteins.

NF-κB promoter activities related to IRF3 were tested using all ZIKV proteins (Fig. 3). Although the expression and downregulation levels were meager, NS2A showed inhibition of NF-κB production by all forms of IRF3. Moreover, NS4A led to decreased NF-κB promoter activities on full-length IRF3 and IRF3 1-390. ZIKV NS1 also acted as an inhibitor of full-length of IRF3 even though the reduction of expression was slight. Levels of NF-κB activation were slightly reduced by NS2A in all forms of IRF3, whereas NS4A showed less reductions and NS1 had no effect.

### ZIKV NS2A or NS4A Inhibited NF-κB Production Activated by MDA5 in a Dose-Dependent and Tag Position-Independent Manner

**Fig. 4:**
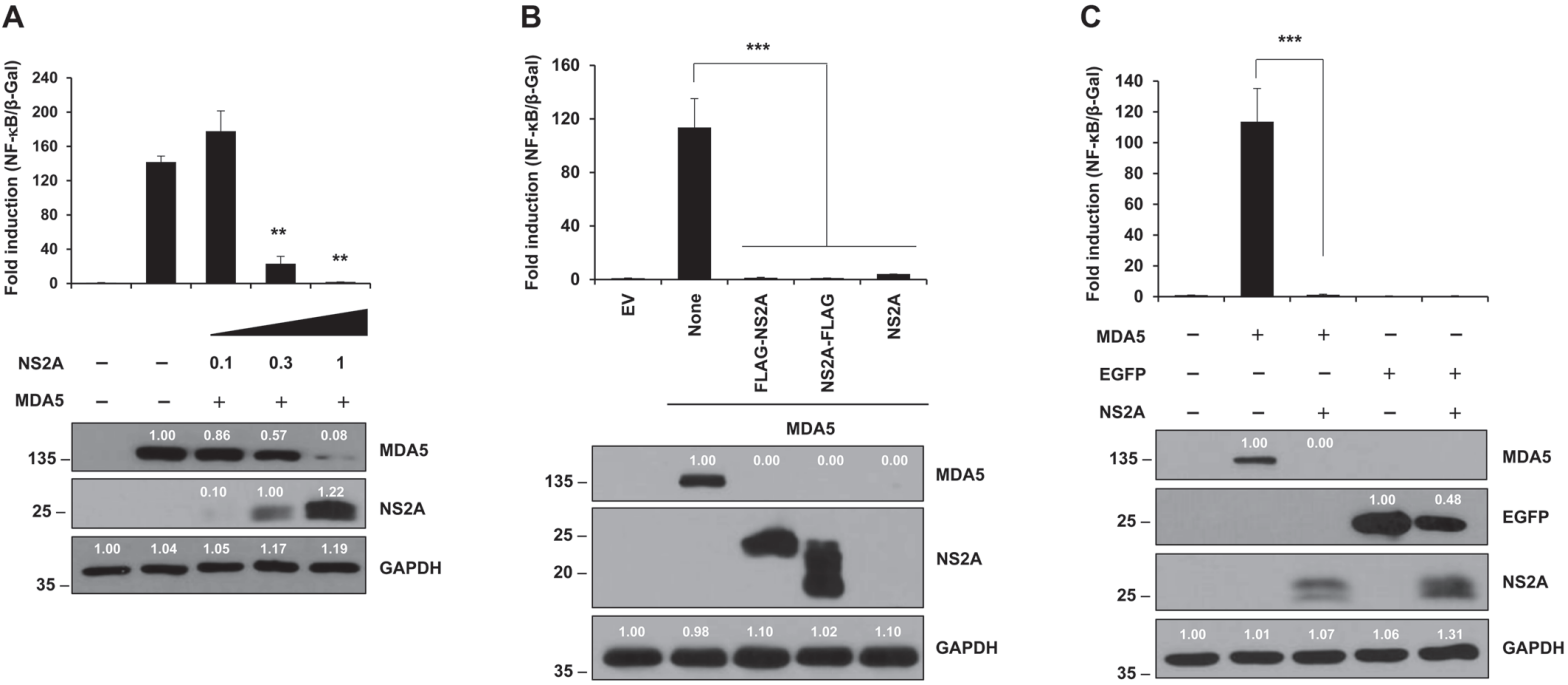
ZIKV NS2A impairs MDA5-induced NF-κB signaling pathway. (**A**) ZIKV NS2A reduced NF-κB promoter activity in a dose-dependent manner (0, 0.1, 0.3, or 1 μg). (**B**) NS2A protein with 3xFLAG-tag on N-terminus or C-terminus and without the 3xFLAG tag showed no different effects on NF-κB productions. (**C**) NS2A protein affected the expression of MDA5 and EGFP proteins. Protein expression was determined by western blotting (bottom panel) with anti-HA antibody (for MDA5), anti-GFP antibody (for EGFP proteins), anti-FLAG antibody (for ZIKV proteins), and anti-GAPDH antibody (for human GAPDH). Results are a representative of two independent experiments. Statistical significance was determined by the Student’s two-tailed *t*-test ***p < 0.001, **p < 0.01, *p < 0.05. The size of the proteins is indicated on the left (kDa).

**Fig. 5:**
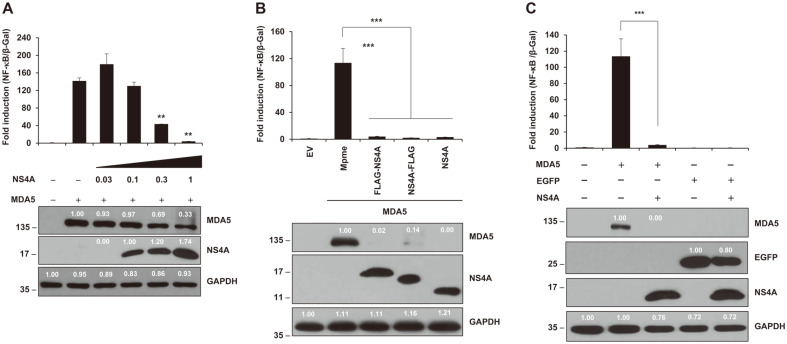
ZIKV NS4A damaged MDA5-induced NF-κB signaling pathway in a dose-dependent manner. (**A**) ZIKV NS4A inhibited NF-κB production by dose-dependent manner (0, 0.03, 0.1, 0.3, or 1 μg). (**B**) NS4A protein with N-terminus or C-terminus 3xFLAG-tag indicated no dissimilar effects on NF-κB productions. (**C**) NS4A protein slightly influenced the expression of EGFP proteins. Protein expression was determined by western blotting (bottom panel) with anti-HA antibody (for MDA5), anti-GFP antibody (for EGFP proteins), anti-FLAG antibody (for ZIKV proteins), and anti-GAPDH antibody (for human GAPDH). Results are a representative of two independent experiments. Statistical significance was determined by the Student’s two-tailed *t*-test ***p < 0.001, **p < 0.01, *p < 0.05. The size of the proteins is indicated on the left (kDa).

Various amounts (0, 0.1, 0.3, or 1 μg) of NS2A-expressing plasmid or (0. 0.03, 0.1, 0.3, or 1 μg) of NS4A-expressing plasmid was transfected into HEK293T cells. Then, luciferase assay for NF-κB promoter activities and western blotting were performed at 24 hpt (Figs. 4A and 5A). Both assays showed that NS2A and NS4A inhibit NF-κB production through down-regulation of MDA5 expression. Furthermore, the tagging systems did not influence NF-κB production in either NS2A- or NS4A-mediated inhibition of NF-kB promoter activities (Figs. 4B and 5B). These results confirmed NS2A and NS4A truly mitigated MDA5-mediated NF-κB production. Finally, we tested whether the MDA5-mediated NF-κB production was mediated by disregulation of cellular transcription and/or translation mechanisms, not by direct interaction with MDA5 by NS2A or NS4A (Figs. 4C and 5C). Interestingly, levels of EGFP protein expression in the presence of NS2A and NS4A were 48% and 80%, respectively, compared to those in the absence of them. This evidence suggested that NS2A and NS4A had a possible negative effect on transcription and/or translation of EGFP protein expression driven by the CMV promoter

## Discussion

In this research, we aimed to show which ZIKV proteins modulate activity of the NF-κB promoter through the RLR (MDA5/RIG-I)-related signaling pathway. Herein, our study showed that ZIKV-encoded NS2A and NS4A proteins strongly antagonize the production of NF-κB by inhibiting most, not all, members of the MDA5/RIG-I signaling pathway with or without changes of the protein expression levels of the components.

The 11-kb RNA genome of ZIKV translated as a single polyprotein, which is proteolytically cleaved later, encodes three structural proteins named C, prM and E, and seven non-structural proteins named NS1, NS2A, NS2B, NS3, NS4A, NS4B and NS5. Both host and viral proteases are involved in the polyprotein cleavage [36]. The C protein builds a nucleocapsid with viral RNA genome, the prM is for virus maturation and egression, and the E protein functions in virus attachment, membrane fusion and entry to host [37]. Nonstructural proteins have multi-functions related to the process of polyprotein, the replication of virus genome and modulation of host immunity. ZIKV NS1 glycoprotein is an antagonist against the innate immune responses [38-40] and a required cofactor for the genome replication and virus morphogenesis. *Flavivirus* NS2A proteins are highly conserved in the family *Flaviviridae* and are invovled in virus replication, assembly, secretion and even suppression of innate immune response [40-47]. NS2B protein is a cofactor to NS3 protein activities functioning for the processing of the polyprotein into an RNA helicase and triphosphatase [48]. NS4A is involved in the arrangement between the host membrane and the virus replication complex [49, 50], and is a well-known interaction inhibitor between RLRs and MAVS [51]. NS4B is one of the factors in the viral replication complex [52]. In addition, both NS4A and NS4B induce cellular dysregulation caused by the disturbance of the Akt-mTOR pathway [53]. NS5 is an RNA-dependent RNA polymerase (RdRp) with a role in viral RNA replication and is also an N-terminal methyltransferase making N-7 and 2'-O methylation of the viral RNA [54-56].

NF-κB is one of the well-known factors in the host immune system against virus infection [16, 17]. After the first recognition of virus RNA genome by PRRs (MDA5 and RIG-I), the host immune system starts to initiate IFN production by activation of MDA5/RIG-I signaling pathways. The secreted IFNs and cytokines lead to activation of NF-κB and subsequent production of pro-inflammatory cytokines [31]. Generally, it is well known that NF-κB pathway is connected to IKKα, IKKβ, IKKγ, IKKβ, IκB, p50 and p65 [57]. However, the non-canonical NF-κB pathways, related to IKKε, TBK1 and IRF3, have only recently been studied [58].

According to our previously published study, ZIKV NS2A or NS4A proteins downregulated all components of the MDA5/RIG-I signaling pathway, with the exception of IRF3-5D which is inhibited only by NS2A but not by NS4A [30]. Similarly, our data

showed ZIKV NS2A and NS4A proteins negatively modulated NF-κB promoter activity when induced by all factors of MDA5/RIG-I signaling pathway, except IKKε and IRF3-5D (Figs. 1-3). Although the ZIKV NS2B-NS3 complex suppresses MAVS and is a mediator of IFN regulatory factor 3 activation (MITA) [32], our results revealed no significant downregulation of NF-κB promoter activities in MAVS by the NS3 and the NS2B-NS3 complex, as with IFN-β production by ZIKV production [30]. NS4A confirmed the possibility to become a candidate universal inhibitor of MDA5-mediated NF-κB signaling. NS4A strongly decreased NF-κB promoter activities via MDA5, RIG-I, RIG-I 1-228 (Fig. 1), MAVS, TBK1 (Figs. 2A and 2B), IRF3-FL and IRF3 1-390 (Figs. 3A and 3B). Even though NS4A inhibited NF-κB production by IRF3-FL and IRF3 1-390 (Figs. 3A and 3B), IRF3-5D (Fig. 3C), which is a mimic of the phosphorylated form of IRF3, is not affected by ZIKV NS4A. These data showed that IRF3 inhibition mediated by NS4A may be caused in a roundabout way and be predicted to be upstream IRF3 kinases such as TBK1 (Fig. 2B), not IKKε. NS4B also displayed inhibition of NF-κB production with two signaling molecules, MDA5 (Fig. 1A) and TBK1 (Fig. 2B). In addition, ZIKV NS1 protein performed as a ‘minor’ inhibitor of the NF-κB activation pathway when induced by RIG-I 1-228 (Fig. 1C) and TBK1 (Fig. 2B). Our study indicated that NS1 has less and selective effect on signaling molecules, which appears to be a clear dissimilarity with previous studies [39, 40]. In our studies, ZIKV NS5 did not suppress any signaling molecules in the NF-κB pathway. Japanese Encephalitis Virus (JEV) NS5 inhibited both IRF3 and NF-κB production [59]; however, in contrast, Dengue virus (DENV) NS5 promotes NF-κB production via NS5 and regulated on activation, normal T cell expressed and secreted (RANTES) [60]. This controversy may suggest that NS5 gene in different viruses may function in different ways. It is interesting that, in most cases, expression of ZIKV proteins led to low levels of signaling proteins and NF-κB promoter activities. However, in some cases, the expression of ZIKV proteins did not match with expression levels of signaling proteins and NF-κB promoter activities. These results suggest that ZIKV proteins may independently influence synthesis or degradation of signaling protein. Fundamental mechanisms are now under investigation.

NS2A and NS4A led to down regulation of NF-κB expressions in a dose-dependent manner (Figs. 4A and 5A). Moreover, the tagging system on the cloning vector did not affect NF-κB production (Figs. 4B and 5B). These data strongly suggest that that NS2A and NS4A down regulated NF-κB production and the inhibition seem to be specifically mediated by NS2A and NS4A proteins themselves. In addition, to check whether the inhibition of NF-κB production and the expression level of MDA5 protein was caused by direct inhibition of the CMV promoter on the vector, we performed luciferase assay and western blot assay using EGFP expression vector (Figs. 4C and 5C). The 52% and 20% reduction of EGFP protein in the presence of NS2A and NS4A, respectively, seem to be less in magnitude compared to the reduction of NF-κB production by NS2A and NS4A. Further examination is undergoing to fathom the mechanisms.

**Fig. 6:**
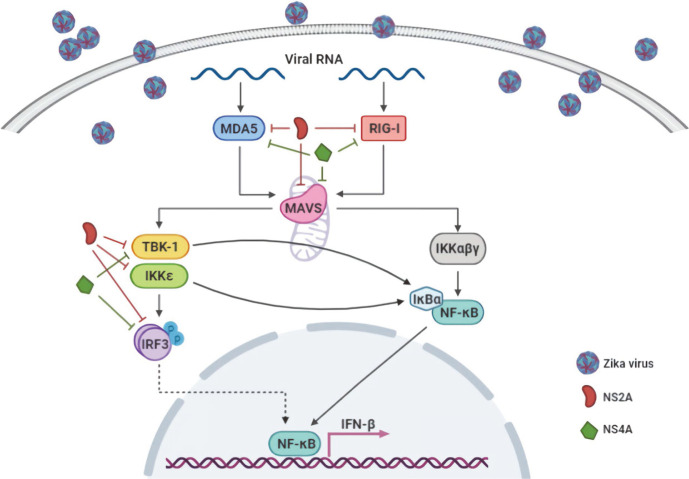
Schematic diagram of MDA-5/RIG-I signaling pathway and its antagonism by ZIKV-encoded NS2A and NS4A. Signaling molecules that are involved in the activation of NF-κB promoter are illustrated. Those molecules, regulated by NS2A and NS4A, are indicated. The schematic diagram was created using biorender.com.

In conclusion, the data presented here demonstrate that ZIKV-encoded proteins seem to be involved in the suppression of NF-κB activation. The most important point of our study is that ZIKV NS2A and NS4A play important roles in the downregulation of NF-κB promoter activity through inhibition of multiple signaling molecules of the MDA5/RIG-I signaling pathway (summarized in Fig. 6). Further studies are currently underway to identify possible mechanisms of NS2A or NS4A in the regulation of the NF-κB promoter activities.
